# Diagnosis and Mobile Application of Apple Leaf Disease Degree Based on a Small-Sample Dataset

**DOI:** 10.3390/plants12040786

**Published:** 2023-02-09

**Authors:** Lili Li, Bin Wang, Yanwen Li, Hua Yang

**Affiliations:** College of Information Science and Engineering, Shanxi Agricultural University, Jinzhong 030801, China

**Keywords:** semantic segmentation, apple leaf, complex environment, small sample dataset, mobile phone recognition system

## Abstract

The accurate segmentation of apple leaf disease spots is the key to identifying the classification of apple leaf diseases and disease severity. Therefore, a DeepLabV3+ semantic segmentation network model with an actors spatial pyramid pool module (ASPP) was proposed to achieve effective extraction of apple leaf lesion features and to improve the apple leaf disease recognition and disease severity diagnosis compared with the classical semantic segmentation network models PSPNet and GCNet. In addition, the effects of the learning rate, optimizer, and backbone network on the performance of the DeepLabV3+ network model with the best performance were analyzed. The experimental results show that the mean pixel accuracy (MPA) and mean intersection over union (MIoU) of the model reached 97.26% and 83.85%, respectively. After being deployed into the smartphone platform, the detection time of the detection system was 9s per image for the portable and intelligent diagnostics of apple leaf diseases. The transfer learning method provided the possibility of quickly acquiring a high-performance model under the condition of small datasets. The research results can provide a precise guide for the prevention and precise control of apple diseases in fields.

## 1. Introduction

There are about 30 kinds of common diseases of apple trees, and there are also more than 10 kinds of disease symptoms in the leaves. It can be observed that apple leaves are the main part of the tree that reflects apple diseases. In practical planting scenarios, orchard managers can determine the type and extent of the disease by visually observing the disease areas on apple leaves. However, the process of determining the type and extent of disease requires significant human and financial resources, making it both time-consuming and laborious, and the results are influenced by several objective factors [1]. The realization of rapid identification of disease types and rapid and accurate diagnosis of disease degrees can provide the basis and technical support for the automatic detection and diagnosis of intelligent agricultural diseases [2,3,4]. Disease warning or precise pesticide spraying should be carried out according to the disease severity to improve the management level, save on manpower and precision medicine, and reduce environmental pollution. However, the accurate segmentation of the lesion area and the leaf area is the key factor that affects the diagnosis of disease severity [5,6].

With the proposed segmentation network models based on deep learning, some researchers have also attempted to apply these segmentation network models to the field of agriculture [7,8,9,10]. The advantage of the semantic segmentation network model in diagnosing the degree of leaf disease is that it can achieve pixel-level classification and calculate the proportion of leaf spot area to leaf area, so that the disease degree can be easily estimated according to the national standard of disease degree diagnosis. For example, Xu et al. [11] used Mask R-CNN to segment leaf images and calculate the number of leaves in the leaf segmentation challenge in 2017, and the method achieved an average accuracy of 89.9% on a segmentation task containing a dataset of 186 images. Esgario et al. [12] proposed a multi-task system based on a convolutional neural network (CNN) to classify different biological stresses of coffee leaves with a simple background and estimate their severity. Later, the author Esgario et al. [13] developed an application based on the smartphone. However, these research methods deal with the segmentation of images with a controlled background (white), so they cannot be applied to images taken in natural scenes. An improved DeeplabV3+ network model was proposed to segment the sweetgum leaf-spot image, where the lesion degree would be graded according to the lesion area. The experimental results showed that the PA, mRecall, and MIoU of the improved DeeplabV3+ network were 94.5%, 85.4%, and 81.3%, respectively, which was superior to the traditional DeeplabV3+, UNet, and SegNet models [14]. The disadvantage is that it does not use the application based on a mobile terminal. Pang et al. [15] proposed an improved RetinaNet model by optimizing the feature pyramid in FPN and the anchor generation strategy to detection wheat spider mites with a wheat field background, and the experimental results showed that the mAP was 81.7%. The disadvantage is that this does not use the diagnosis of the disease degree.

These studies show that semantic segmentation of plant disease spot images based on the deep learning semantic segmentation model is ideal. The advantage of disease severity diagnosis based on the semantic segmentation network model is that it does not have strict requirements for the disease severity distribution in the training disease data set used; that is, there is no need for images of all levels of disease severity in the training data set. After the lesion area is segmented, the disease severity can be easily calculated according to the diagnosis standard of disease severity. The application had two functions: one was to estimate the severity of the damage location based on semantic segmentation, and the other was to identify the category of the damage segmentation area. Therefore, the diagnosis of apple leaf disease severity based on the semantic segmentation network model is worth studying.

Several studies have recently been conducted in recognizing apple leaf disease images and achieved good results [16,17,18]. However, few studies have been carried out on the segmentation of apple leaf lesion areas and diagnosis of disease severity based on the idea of semantic segmentation. Chao et al. [19] proposed the use of the full convolution semantic segmentation network model based on an encoder and decoder to recognize four common diseases of apple leaves. In addition, the end-to-end pixel classification of the apple leaves’ diseased spot area, healthy leaf area, and background area were realized, but the disadvantages were that the study objects did not include cases of multiple diseases in a single leaf image, and the severity of the disease was not diagnosed.

Although the studies mentioned above realized the recognition of apple leaf disease or segmentation of disease spots and achieved good results, they did not complete the application of the mobile terminal.

Driven by these deficiencies, the main novel aspects of this work include:

(1) The research object of this work not only includes single diseases in a single leaf image, but also multiple diseases in a single leaf image.
(2) In this study, the DeepLabV3+ network was used to achieve accurate segmentation of the lesion area and calculation of the disease severity.
(3) A mobile application is finished based on the DeepLabV3+ network.

## 2. Materials and Methods

### 2.1. Image Dataset

#### 2.1.1. Image Dataset Acquisition

**Figure 1:**
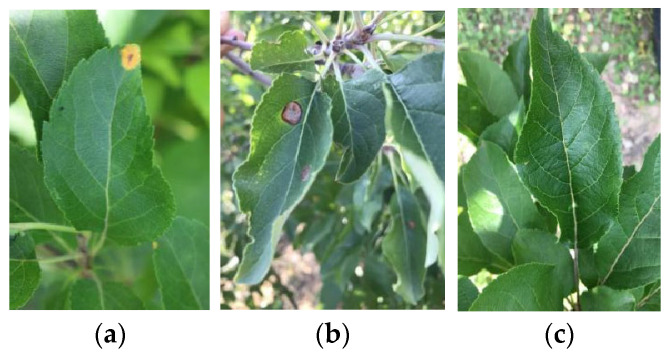
Example of apple leaf images: (**a**) rust; (**b**) ring rot; (**c**) healthy.

The diseased apple leaf images were collected using smartphones (iPhone13) under real conditions from the planting base of the Pomology Institute of Shanxi Agricultural University and farmers’ orchards, and the resolution of the images was 3024 × 4032. The original dataset was prepared with 267 images, which included 152 images of ring rot and 115 images of rust. These images were captured with uneven illumination intensities in heterogeneous fields and in wild scenarios. All the images collected in this paper are defined according to their disease categories and have been saved in JPEG format. Some examples of images include rust, ring rot, and healthy leaves, as shown in Figure 1.

#### 2.1.2. Dataset Calibration

**Figure 2:**
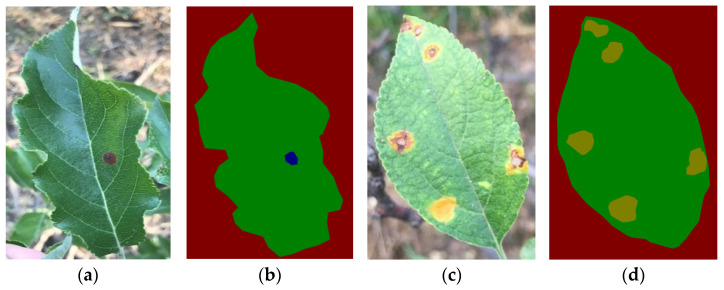
Examples of labeled disease images: (**a**) images of ring rot; (**b**) images of ring rot after labeling; (**c**) images of rust; (**d**) images of rust after labeling.

The two disease datasets were calibrated using polygons of the annotated Colabeler V2.0.4, and the leaf area, the diseased spot area, and the background area were labeled. The annotated data set was divided into the training set, validation set, and test set according to the ratio of 6:2:2. The data from the training set and the validation set were enhanced to three times the original, and the enhanced images included 477 training sets, 162 verification sets, and 54 test sets, respectively. Image enhancement was mainly horizontal [20], vertical, and rotated. Figure 2 shows an example of an image in the dataset and its calibration effect.

Although the deep learning neural network is very powerful, if there are not enough images, it will result in overfitting, and the desired results will not be achievable [21]. Many researchers have conducted significant work on this topic. Therefore, in order to avoid over-fitting, data augmentation of the training set and validation set was carried out. The data distributions of the original dataset, augmented dataset, training set, validation set, and test set are shown in Table 1.

### 2.2. Semantic Segmentation Network Model

In this section, we describe in detail the semantic segmentation convolutional neural network model, DeepLabV3+, which we used in our work.

#### 2.2.1. DeepLabV3+ Network Model

**Figure 3:**
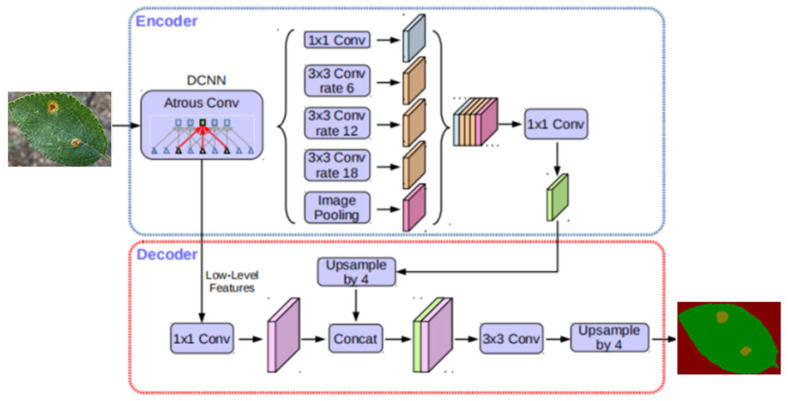
Schematic diagram of the overall structure of the DeepLabV3+.

The DeepLab series is a series of semantic segmentation algorithms proposed by the Google team, mainly including DeepLabV1, DeepLabV2, DeepLabV3, and DeepLabV3+. Among them, DeepLabV1 combines with CNN and adopts DenseCRFs as a post-processing method to improve the boundary clarity of semantic segmentation results [22]. Compared with DeepLabV1, DeepLabV2 is different in that it proposes the Atrous Spatial Pyramid Pooling (ASPP) module, which is used to combine the feature maps generated by the convolution of cavities with different cavity rates. Richer context information is obtained and the segmentation accuracy is improved. On the other hand, DeepLabV3 optimizes the ASPP module based on DeepLabV2, adding 1 × 1 Convolution and BN operations to increase the global context information. In order to improve the segmentation accuracy of object boundaries, DeepLabV3+ adds a decoder module based on DeepLabV3 to improve the segmentation effect of network models. The structural framework of the DeepLabV3+ network is shown in Figure 3.

**Figure 4:**
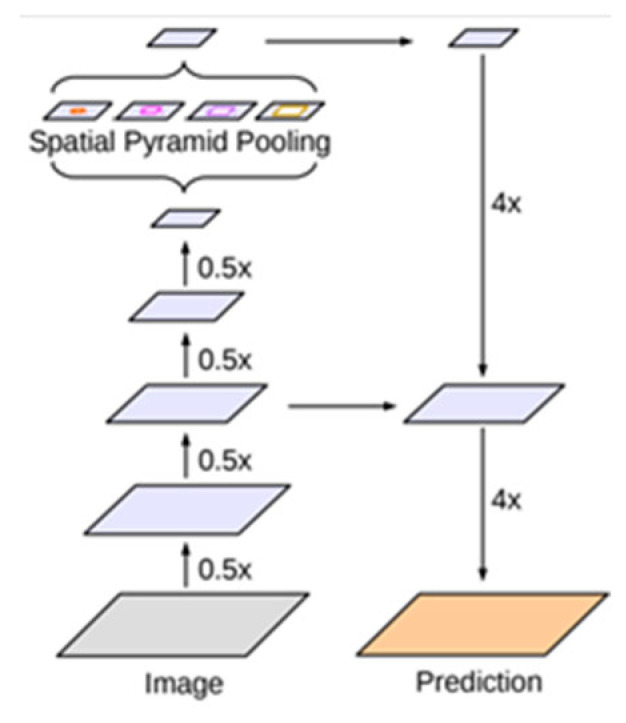
Encoder–decoder module used in DeepLabV3+.

Figure 3 shows the structure diagram of the DeepLabV3+ network model, and it can be seen from the figure that an encoder–decoder module was introduced into the DeepLabV3+ network model. From Figure 4, we can see that the main body of the encoder is DCNN, with Atrous convolution, which can use common classification networks such as ResNet, as well as the Atrous Spatial Pyramid Pooling (ASPP) with Atrous convolution. The purpose of adding Atrous convolution with different dilation rates is to extract context information of different scales. And in the decoder, through adding lateral connection, low-level features are fused with encoded high-level features to increase the spatial information in order to improve the segmentation accuracy of the model.

**Figure 5:**
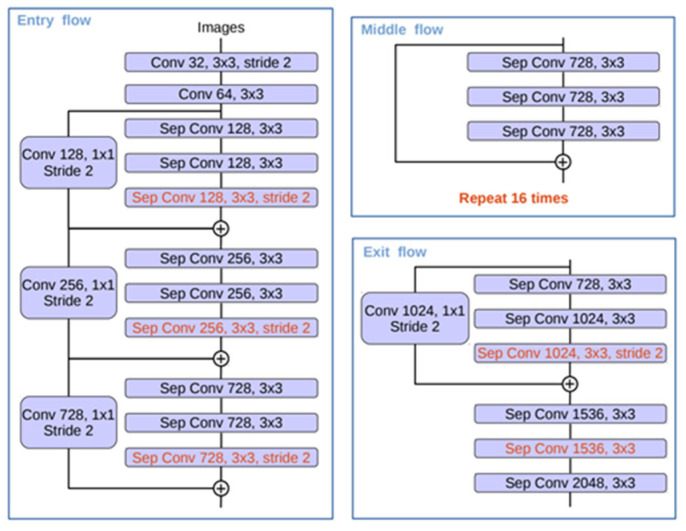
The improved Xception network.

Moreover, an improved Xception is used as the backbone of the DeepLabV3+ network to improve segmentation speed and accuracy of the network. The improved Xception network is shown in Figure 5. Improvements to Xception mainly include the following aspects: (1) Entry flow remains the same, but more middle flow is added. (2) All max pooling layers are replaced by depthwise separable convolutions with stride = 2. (3) Batch normalization and ReLU operations are added after each 3 × 3 depthwise convolution.

#### 2.2.2. Model Evaluation Indicators

The semantic segmentation model is mainly evaluated in terms of pixel accuracy (PA), mean pixel accuracy (MPA), and mean intersection over the union (MIoU) [23]. As semantic segmentation ends up studying a classification task, thus, its predictions often contain the four cases of true positive (TP), false positive (FP), true negative (TN), and false negative (FN).

Suppose there are *k+1* categories (which contain a background category), and the pixels of $\;P_{ij}\;$ representing category *i* are identified as category *j*, which is FP; the pixels of $\;P_{ji}\;$ representing category *j* are identified as category *i*, which is FN; and the pixels of $P_{ii}\;$ representing category *i* are identified as category *i*, which is TP.

(1) Pixel accuracy (PA): This is the simplest index of image segmentation. It refers to the proportion of the number of correctly classified pixels of the current category in the total number of pixels. As shown in Equation (1):

(1) $$\mathrm{PA}=\frac{\sum_{i=0}^{k}P_{ii}}{\sum_{i=0}^{k}\sum_{j=0}^{k}P_{ij}}$$

(2) Mean pixel accuracy (MPA) is the average value of all types of pixel accuracy, and its calculation method is shown in Equation (2):

(2) $$\mathrm{MPA}=\frac{1}{k+1}\overset{k}{\underset{i=0}{\sum }}\frac{P_{ii}}{\sum_{j=0}^{k}P_{ij}}$$

(3) Mean intersection over union (MIoU) is the most common metric in semantic segmentation [24], and is calculated based on classes. Specifically, the IoU of each class is calculated, and then the calculated IoU of all classes is accumulated and, finally, averaged. The specific calculation is shown in Equation (3):

(3) $$\mathrm{MIoU}=\frac{1}{k+1}\overset{k}{\underset{i=0}{\sum }}\frac{P_{ii}}{\sum_{j=0}^{k}P_{ij}+\sum_{j=0}^{k}P_{ji}-P_{ii}}$$

### 2.3. Experiment Setup

The experiments were conducted in the environment of Ubuntu18.04, with Intel Core i9 9820X, 64G memory, GeForce RTX 2080Ti 11G DDR6, using the deep learning framework TensorFlow and Cuda10.1 for training.

## 3. Experiments and Analysis

**Figure 6:**
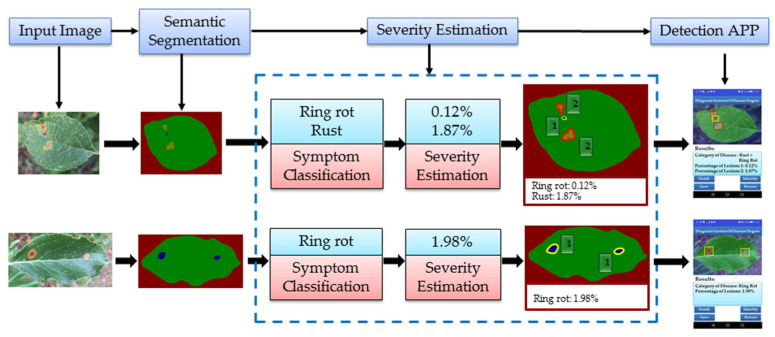
Research framework.

In this study, the method of combining the disease spot segmentation with the disease spot degree calculation was proposed to diagnose the disease grade of the apple leaves. There were two main stages: in the first stage, semantic segmentation network models (GCNet, PSPNet, and DeepLabV3+) were used to segment the diseased spot region and leaf region. In the second stage, the area of the disease spot and the area of the leaf were calculated, and the degree of the disease and its classification grade was estimated. The proposed architecture is shown in Figure 6.

### 3.1. Comparison of Average Segmentation Accuracy of Different Models

This section used three models—DeepLabV3+, PSPNet [25], and GCNet [26]—for the segmentation task of apple leaves as well as diseased areas. The optimizer used in the training was Adam, the epoch was 50, the initial learning rate was 0.001, the backbone network used ResNet50 [27], and the batch size was 4. The training strategy based on transfer learning was applied to the segmentation network [28]; that is, the weight values of the model were initialized by using the weight parameters of the model that was pre-trained on the ImageNet dataset [29], and the parameters in the feature extraction stage were not updated during the training process. If the training loss value did not decrease for two consecutive epochs in the training process, the learning rate was automatically adjusted to 1/2 of the original value. At the same time, the early stopping mechanism strategy was used in the training process; that is, if the loss function value of the training set did not decrease within 10 epochs, the training of the model was ended.

To analyze the segmentation performance of the three models on the apple leaf disease dataset for this experiment, Table 2 lists information on the hyper-parameters, number of parameters, segmentation speed (single image segmentation time), MIoU, MPA, and Kappa coefficients used by the models.

It can be seen from Table 2 that the three classical segmentation network models obtained good results for the MPA, MIoU, and Kappa coefficients in the apple leaf disease segmentation task. By comparing different learning rates of the same model, it was found that the segmentation effect of the model was better when the learning rate was set to 0.001, compared with that when the learning rate was set to 0.01.

When the learning rate was set to 0.001, by comparing the MPA, MIoU, and Kappa coefficients of the three network models, the analysis showed that the DeepLabV3+ network model had the best segmentation effect, PSPNet was the second-best, and GCNet had the worst segmentation effect. Considering the number of model parameters and the speed of segmentation, PSPNet had more than twice the number of parameters as DeepLabV3+, while the MPA and MIoU of the PSPNet were 0.8 and 2 percentage points lower than DeepLabV3+, respectively. Additionally, when the learning rate was 0.01, DeepLabV3+ had the best segmentation effect, PSPNet had the second-best segmentation effect, and GCNet had the worst segmentation effect.

**Figure 7:**
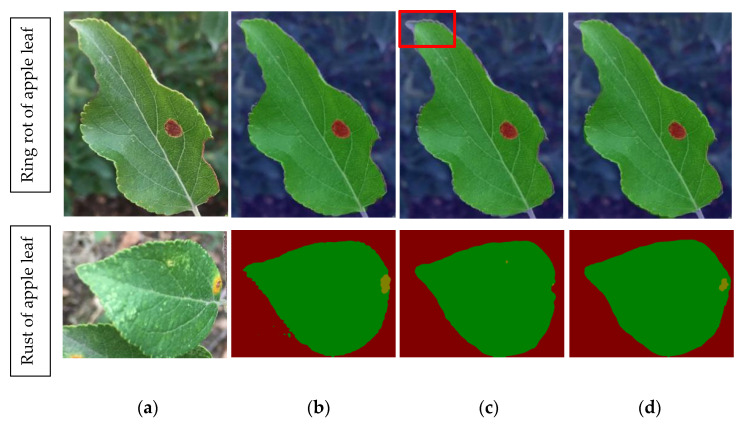
Segmentation effect of different models: (**a**) original image; (**b**) DeepLabV3; (**c**) GCNet; (**d**) PSPNet.

In summary, it can be found that the DeepLabV3+ showed a better segmentation performance than the other two segmentation network models, PSPNet and GCNet. The segmentation results of three semantic segmentation network models for different diseases are shown in Figure 7.

It can be seen from Figure 7 that the segmentation effect of DeepLabV3+ and PSPNet network models on ring rot leaf was not significantly different, and both of them were able to correctly segment leaves and disease spots, although the segmentation effect of GCNet on leaves was slightly worse (the part framed by the red line in Figure 7c). However, for the segmentation of rust, DeepLabV3+ had the best effect on the segmentation of the diseased spot, almost able to correctly segment the entire diseased spot. The PSPNet had the second-best segmentation effect, and only part of the lesions was segmented correctly. However, GCNet had the worst segmentation effect for apple rust and almost failed to segment the spots correctly.

### 3.2. Comparison of Segmentation Accuracy of Different Disease Types

To further compare and analyze the segmentation performance of the three semantic segmentation network models, the pixel accuracy (PA) and the intersection over union (IoU) for each of the four classes (rust, ring rot, leaf, background) of the three network models were compared, and the results are shown in Table 3. Those marked in bold in the table are the best segmentation network models corresponding to the metrics in the current segmentation category.

As can be seen from Table 3, except for the segmentation of the apple leaf rust category, the DeepLabV3+ model achieves the best segmentation index for other categories of IoU compared with the other two network models. Apart from the category of leaf, the PA index of the DeepLabV3+ was higher than that of the PSPNet and GCNet models in other disease categories. It can be seen that the performance of the IoU index changes with the change in the PA index in general, but they were not related. According to Table 2 and Table 3, compared with other network models, the DeepLabV3+ had higher segmentation PA performance indexes in most categories, and the MPA of the DeepLabV3+ network was also slightly higher than that of the PSPNet.

### 3.3. Comparison of Model Convergence Performance

**Figure 8:**
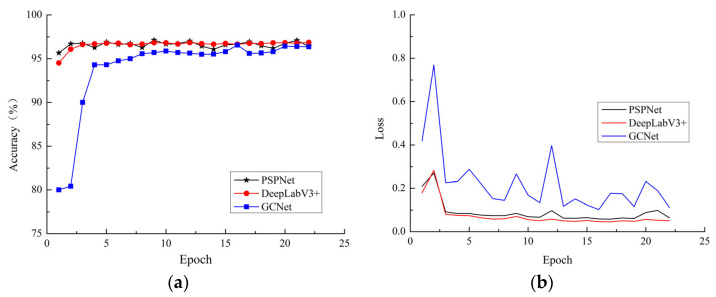
Convergence of different models: (**a**) variation of pixel accuracy with training rounds; (**b**) variation of validation set loss values with training rounds.

To compare and analyze the convergence performance of three semantic segmentation network models (DeepLabV3+, PSPNet, and GCNet) on the verification set, the curve of pixel accuracy and loss value of the three semantic segmentation network models on the validation set with the change of training times is shown in Figure 8.

The initialization of the weights in the model training was performed by loading the pre-trained weight parameters of the backbone network on the ImageNet dataset directly into the model for training. Therefore, it can be seen from Figure 8 that due to the use of transfer learning, the convergence speed and the starting point of convergence of the three semantic segmentation network models were very high. The convergence starting points of DeepLabV3+ and PSPNet were both close to 95%. The GCNet network had the worst convergence performance, while DeepLabV3+ and PSPNet had the same convergence performance. It can be seen that the MPA of the DeepLabV3+ model was slightly higher than that of PSPNet, and the overall MIoU of the DeepLabV3+ network model was the highest. Comprehensive analysis shows that the performance in segmentation of apple leaf diseases based on the DeepLabV3+ network model was the best.

### 3.4. Hyper-Parameter Comparison of the DeepLabV3+ Network Model

Table 4 shows the segmentation performance comparison results of the DeepLabV3+ network model under different learning rates and different optimizer combinations.

As can be seen from Table 4, when different initial learning rates and optimizers were chosen, the difference between the MPA values was less than one percentage point, with a maximum value of 97.26% and a minimum value of 96.34%. However, there was a significant difference among the MIoU, with the maximum value being 83.85% and the minimum value being 80.42%. This indicates that the selection of hyper-parameters (learning rate, optimizer) had a certain impact on the segmentation performance of DeepLabV3+. When the DeepLabV3+ model selected the same optimizer, the MPA obtained by the model under the condition of a 0.001 learning rate was higher, and the MPA values were 97.21% and 97.26%, respectively. When the DeepLabV3+ model chooses the same learning rate, the model could obtain higher MPA, MIoU, and Kappa values when using the SGD optimizer rather than the Adam optimizer. Through comparative analysis, it can be concluded that when SGD was selected as the optimizer and the initial learning rate was set to 0.001, DeepLabV3+ was able to achieve better segmentation performance, and its MPA, MIoU, and Kappa values were 97.26%, 83.85%, and 0.9781, respectively.

Based on the above analysis, in the fine-tuning training stage of the model, the performance of the trained model was better when the initial learning rate was set to a low value. The reason was that in the transfer learning mode, the front-end layers of the network are well-trained, and the weight parameters of the model are close to the optimal solution. Using a higher learning rate in the fine-tuning training phase could easily lead to the model skipping the optimal solution and generating larger oscillations, resulting in higher loss values and reduced accuracy.

### 3.5. DeepLabV3+ Backbone Network Selection

Based on the analysis in the previous section, the effects of different backbone networks on the performance of the semantic segmentation model DeepLabV3+ in apple leaf spot segmentation were compared through experiments. Table 5 shows the segmentation performance comparison of different backbone networks, ResNet18, ResNet50, and MobileNetV3, selected by DeepLabV3+ [30]. Among them, the hyper-parameters adopt the learning rate (0.001) and optimizer (SGD) which were optimized previously, in Section 3.4.

It can be seen from Table 5 that when ResNet 50 was selected as the backbone network, the speed of segmentation of the DeepLabV3 + model was 75.46 ms and the MPA and MIoU indexes were the highest, at 97.26% and 83.85%, respectively. However, due to the simple structure of the MobileNetV3 network, the MPA and MIoU of the segmented network model based on MobileNetV3 as the backbone network were at their lowest levels, and the MIoU index was as low as 50.30%. The segmentation network model DeepLabV3+, with ResNet18, ResNet50, and MobileNetV3 as the backbone network, did not differ significantly in terms of segmentation time, and the results were all at the same level.

### 3.6. Disease Degree Diagnosis

The degree of apple leaf disease varies with the proportion of diseased spot area. According to the relevant standards of the Quality of Operation of Air assisted Orchard Sprayers (NY/T 992-2006) issued by the Ministry of Agriculture and Rural Affairs of the People’s Republic of China, the diagnostic criteria for the disease degree of apple leaves in this study were determined and the disease grades were classified. Specific classification standards are shown in Table 6.

The image semantic segmentation method was used to segment the leaf area and the lesion area of the apple disease leaf, the proportion of the total number of pixels in the diseased spot area to the whole leaf area was calculated, and the disease degree of the apple leaves was obtained according to the disease degree classification standard. The specific calculation is shown in Equation (4).

(4) $$DD=\frac{S_{d}}{S_{d}+S_{h}}$$

where DD is the diagnosis result of the current leaf disease degree, $S_{d}$ is the total number of pixels in the diseased area obtained by segmentation, and $S_{h}$ is the total number of pixels in the healthy area of the apple leaf.

**Figure 9:**
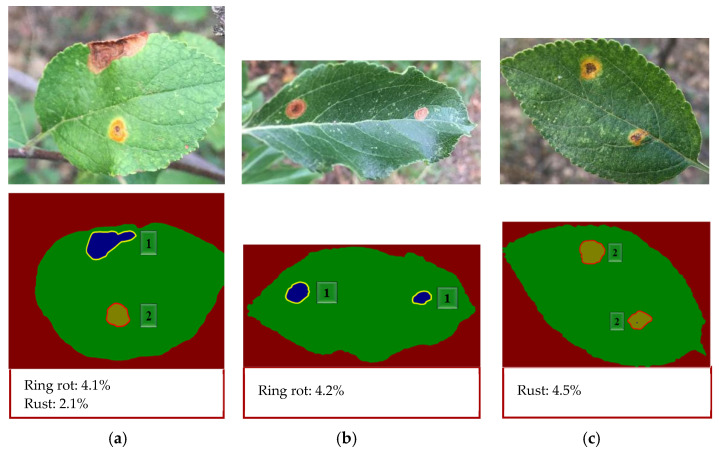
The segmentation effect of different diseases: (**a**) ring rot and rust of apple leaf; (**b**) ring rot of apple leaf; (**c**) rust of apple leaf.

Figure 9 shows the results of the semantic segmentation network model DeepLabV3+ in symptom classification and severity estimation, with ResNet50 as the backbone network, SGD as the optimizer, and 0.001 as the initial learning rate.

It can be observed from Figure 9 that satisfactory results were achieved in the semantic segmentation stage, which correctly classified and segmented lesion and leaf regions in most cases. Figure 9 shows the extent of the disease, calculated from the segmentation results, and the disease grade, determined by comparison with Table 6. The results show that this method was effective and feasible for disease recognition and disease degree estimation of diseased apple leaves photographed with a complex field background.

### 3.7. Apple Leaf Disease Detection System Based on an Android Mobile Phone

#### 3.7.1. The Design of the APP

**Figure 10:**
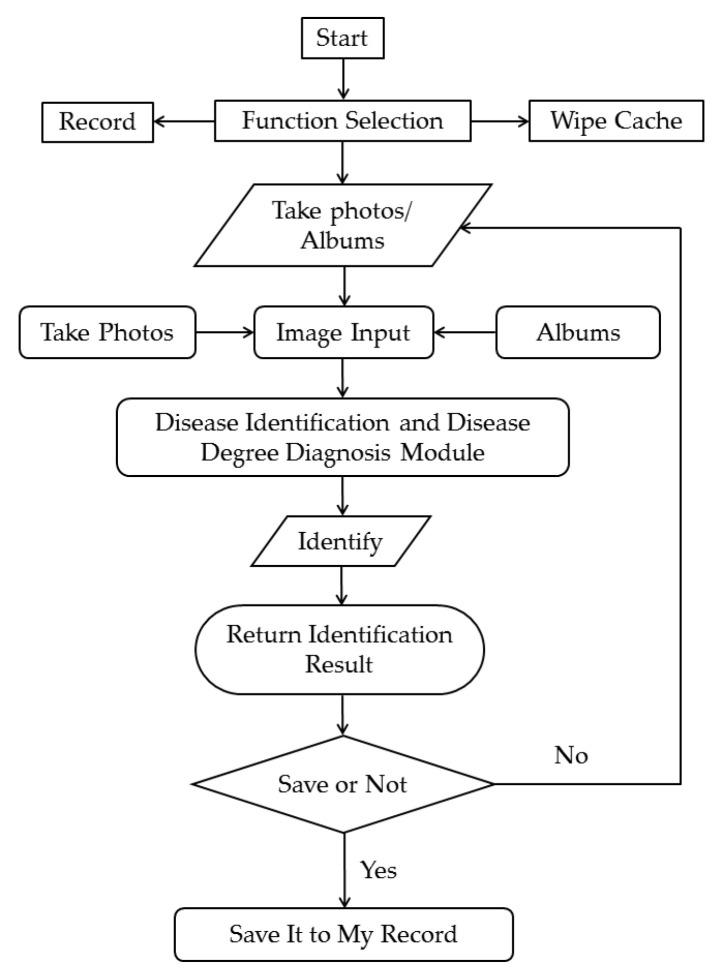
The flow chart of the system.

To verify the effect of the proposed method in practical application, the developed mobile terminal identification system was used. The workflow of the system is shown in Figure 10.

#### 3.7.2. The Implementation of the App

According to the above system design process, in order to realize the identification and detection of apple leaf diseases by the Android app, work needs to be done: (1) Prepare two files, libtensorflow_inference.so and libandroid_tensorflow_inference_java.jar. (2) Convert the trained model weight file (.ckpt file) into a .pb file. (3) Complete the implementation of the Android system.

**Figure 11:**
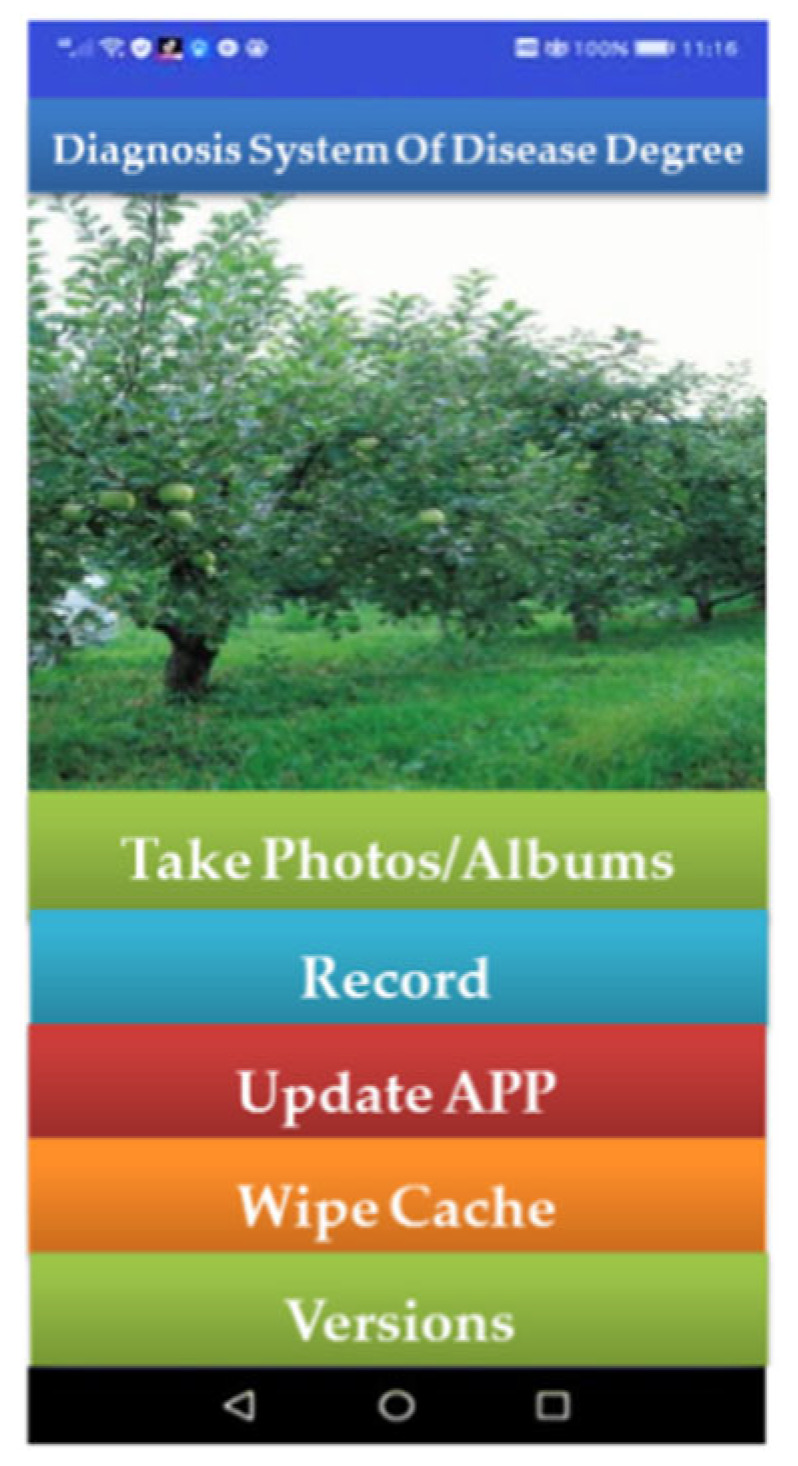
The main interface of the app.

Install the app by copying or downloading the program file with the .APK to an Android device. The concrete implementation of this process is as follows: firstly, the trained .ckpt model is converted to a .pb file so that its parameters can be used in Android Studio. Secondly, use the two code files provided by the official website to put the previously saved .pb file into the directory of app/src/main/assets in the Android project, and then put the two code files (.jar and .so files) in the directory of app/libs and app/libs/armealbi-v7a, respectively. Finally, call the model to complete the configuration. The main interface of the system is shown in Figure 11.

**Figure 12:**
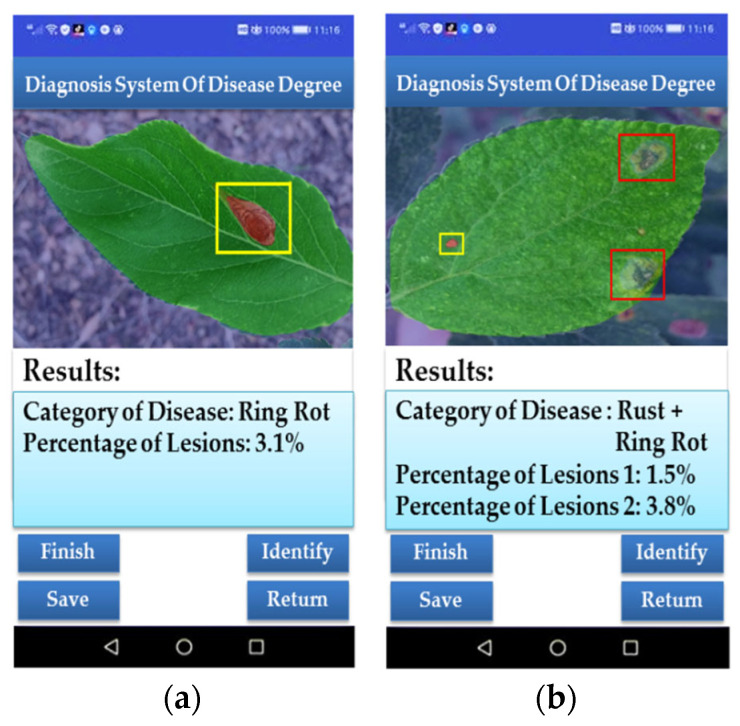
The results of APP program recognition: (**a**) the result of a single disease per leaf; (**b**) the result of multiple diseases per leaf.

The users can obtain target images by taking photos or obtaining them from the local image library. After taking photos or uploading photos, the identification button on the main interface will allow the users to access the developed disease degree diagnosis system app [31]. The disease detection and identification results will be obtained through the semantic segmentation network model DeepLabV3+, which will be embedded in the application, and the APP interface will jump from the current interface to the detection results interface. The marking of the diseased area, the results of disease identification, and the proportion of diseased spots will be displayed in the detection result interface. In this study, a Huawei P20 mobile phone was used to test the system. After testing, it was found that it took about 44 s to process per disease image. The time required to upload the image to the server was more than 35 s, while the time to process the image was less than 10 s, which indicates that the system can be used for apple leaf disease detection with the current 4G network. The test results are shown in Figure 12.

## 4. Discussion

In the context of this work, image-based object detection and classification applications based on deep learning are promising in their ability to revolutionize many avenues of research.

The method proposed in this paper was mainly aimed at the detection of apple rust by photographs with complex backgrounds and the determination of the disease grade. Although the MPA and MIoU of the proposed model reached 97.26% and 83.85%, respectively, the average detection time of the model deployed on the mobile terminal was 44 s. This study provides a guiding method for the detection of apple leaf diseases in a complex environment. However, there are still some problems that need to be improved, such as the existence of false detection, missing detection, false segmentation, and difficulty with the segmentation of multiple apple leaves in the same image. Although the dataset used in this paper was collected considering different onset periods, different light conditions, and different growth periods, and data enhancement and transfer learning techniques were adopted to improve the accuracy of disease identification and lesion segmentation of the model to a certain extent, the category datasets had poor diversity, with few samples. Therefore, a more diversified and reliable disease dataset can be established by various methods in the future, such as collecting leaves with different disease severities in different growing environments and using new data expansion methods (by introducing segmentation modules with attention mechanism into CycleGAN) so as to finally achieve the purpose of improving the segmentation effect of the network model.

## 5. Conclusions

In this work, three classical semantic segmentation network models (DeepLabV3+, PSPNet, and GCNet) were used to complete the segmentation task of apple leaves and lesion areas based on the collected apple leaf images, which consisted of both healthy leaves and leaves with two categories of diseases, with complex backgrounds. Due to the limited dataset, this study chose the transfer learning method to fine-tune the parameters of the model. In the process of this experiment, the segmentation performance of the model was compared and analyzed. In addition, the effects of different hyper-parameters, such as learning rates, optimizers, and the backbone of the DeepLabV3+ network model, on the performance of the model were discussed. Finally, the classification diagnosis of apple leaf disease was examined. Our conclusions are as follows:

1. The segmentation performance and convergence performance of three semantic segmentation network models (DeepLabV3+, PSPNet, and GCNet) for apple leaf and spot regions were compared and analyzed. The results show that the DeepLabV3+ network model has better segmentation performance than the other two network models (PSPNet and GCNet), and the three network models all show that the effect when using a learning rate of 0.001 is better than that when using 0.01.
2. The optimal combination of hyper-parameters and the selection of a backbone network for the DeepLabV3+ network model were studied. The results show that the optimal hyper-parameters of the DeepLabV3+ network model are a set learning rate of 0.001, optimizer SGD, and MPA and MIoU values of the model that reach 97.26% and 83.85%, respectively. By comparing the performance of the semantic segmentation network model DeepLabV3+ with the three backbone networks, it can be concluded that ResNet50 has the best comprehensive performance. The MPA and MIoU values of the segmentation model reached 97.26% and 83.85%, respectively.

In this paper, it was demonstrated that the proposed method can overcome the effect of a complex background on image segmentation, and fine segmentation of leaf lesions and edges of different apple trees can be achieved. The network model’s segmentation precision was high, and the disease degree diagnosis was accurate. In addition, the model can realize real-time segmentation of the disease spot. This study laid a foundation for the accurate identification of apple tree disease types and the accurate diagnosis of disease degree.

## Figures and Tables

**Table 1:** The data distributions.

Classes	Original Dataset	Training Set	Validation Set	Augmented Dataset	Test Set
Rust	115	69	23	276	23
Ring rot	152	90	31	363	31
Total	267	159	54	639	54

**Table 2:** Segmentation performance of the models.

Model	Initial Learning Rate	Parameter Quantity (M)	Split Speed (ms/Picture)	MPA (%)	MIoU (%)	Kappa Coefficient
DeepLabV3+	0.001	39,120,996	75.43	97.21	83.40	0.9721
PSPNet	0.001	67,904,616	75.5	96.47	81.32	0.9806
GCNet	0.001	49,677,289	75.43	96.41	80.19	0.9720
DeepLabV3+	0.01	39,120,996	75.44	96.34	80.42	0.9721
PSPNet	0.01	67,904,616	75.54	96.26	77.62	0.9725
GCNet	0.01	49,677,289	75.44	94.40	77.97	0.9704

**Table 3:** PA and IoU indicators of different models.

Evaluation Indicators	Network Model	Background (%)	Leaves (%)	Rust (%)	Ring Rot (%)
IoU	DeepLabV3+	**93.89**	**94.51**	71.35	**73.86**
	PSPNet	93.29	93.67	72.99	60.83
	GCNet	93.24	93.83	68.88	69.35
PA	DeepLabV3+	**96.63**	97.28	**89.2**	**84.67**
	PSPNet	95.46	**97.75**	86.2	66.97
	GCNet	95.94	97.39	80.39	75.55

**Table 4:** Performance of DeepLabV3+ model.

Model	Initial Learning Rate	Optimizer	MPA (%)	MIoU (%)	Kappa Coefficient
DeepLabV3+	0.01	Adam	96.34	80.42	0.9588
DeepLabV3+	0.001	Adam	97.21	83.40	0.9721
DeepLabV3+	0.01	SGD	97.11	83.72	0.9767
DeepLabV3+	0.001	SGD	97.26	83.85	0.9781

**Table 5:** DeepLabV3+ with different backbone networks.

Backbone Network	Parameter Quantity (M)	Split Speed (ms/Picture)	MPA (%)	MIoU (%)
ResNet18	15,347,044	75.35	95.07	74.50
ResNet50	39,120,996	75.46	97.26	83.85
MobileNetV3	699,726	75.41	89.55	50.30

**Table 6:** Grading table of disease spots on apple leaves.

Disease Grade	Classification Standard of Leaf Disease
0	No disease
1	The lesion area accounts for less than 10% of the whole leaf area
3	The lesion area accounts for 11~25% of the whole leaf area
5	The lesion area accounts for 26~40% of the whole leaf area
7	The lesion area accounts for 41~65% of the whole leaf area
9	The lesion area accounts for more than 65% of the whole leaf area

## Data Availability

Not applicable.
